# Examining the Relationship Between Differing Implementation Patterns of an Evidence-Informed Frontline Nursing Care Model and Implementation Success in a National-Level Study

**DOI:** 10.1155/jonm/9652060

**Published:** 2025-10-28

**Authors:** Miriam Bender, Maricela Cruz, John David Coppin, Marjory Williams

**Affiliations:** ^1^Sue & Bill Gross School of Nursing, University of California Irvine, Nursing & Health Sciences Hall, Room 4317, Irvine, California, USA; ^2^Biostatistics Unit, Kaiser Permanente Washington Health Research Institute, Kaiser Permanente, 1730 Minor Ave, Ste. 1600, Seattle, Washington, DC 98101, USA; ^3^Central Texas Veterans Health System, 1901 Veterans Memorial Dr, Temple, Texas 76504, USA

**Keywords:** Bayesian analysis, clinical nurse leader, effectiveness, implementation, model validation, nursing care delivery, positive work environment, system intervention

## Abstract

**Introduction:**

The Clinical Nurse Leader (CNL) care model, which is increasingly being adopted by health systems across the United States and abroad, is a different way of organizing frontline nursing care delivery, in contrast to traditional “staff nurse ratio” models. However, variability in implementation and outcomes has been noted across health settings.

**Aim:**

A psychometrically validated CNL care model survey instrument operationalizes a complex implementation pathway that leads to improved care quality outcomes. The purpose of this study was to identify implementation patterns in 66 clinical units across 9 hospitals in 5 states and examine their relation to implementation success.

**Methods:**

The survey was administered between 2016 and 2019 to a national sample of administrators/clinicians involved in CNL care model implementation. The survey measures presence of the five domains of the model and implementation success. We analyzed the complex hierarchical structure of the data using a Bayesian framework.

**Results:**

1265 participants responded, and 1223 (97%) provided success and rating scores. The analysis estimated CNL care model elements best discriminating between lower and higher implementation success. Outcome domain survey item scores were most consistently predictive of implementation success. In terms of Readiness and Structuring and CNL Practice, discrete patterns emerged, suggesting efficacious pathways toward improved care environments and care quality.

**Conclusions:**

Findings describe specific CNL care model organizational readiness and unit-level workflow implementation patterns that drive CNL practices and the production of expected outcomes, including positive work environments. The research provides actionable implementation and outcome evidence about a different way to organize nursing knowledge and practice into a care model that can be successfully adopted within real-world healthcare settings to achieve improved care environments and higher quality patient care.

## 1. Introduction

A significant number of patients are harmed or die every year because of unsafe, inappropriate, or inadequate healthcare delivery [1]. The Agency for Healthcare Research and Quality (AHRQ) has long identified Registered Nurses (RNs) as a patient safety strategy for reducing patient mortality and morbidity [2]. However, variability in research findings indicate the relationship is not as simple as “more nurses = better outcomes” [3, 4]. Despite the fact that RNs are the largest health workforce component [5], with identified potential and capacity to improve patient care quality and safety [6, 7], it is disconcerting to conclude that the literature does not currently provide strong evidence supporting any well-specified frontline model of nurse care delivery [8]. Because of this, the relationships between specified RN actions and outcomes in the context of value-based healthcare remain ambiguous [9]. Knowledge elucidating RNs' direct and indirect mechanisms of action as organized within robustly conceptualized frontline models of care is needed, including evidence linking these actions to improved care quality and safety [10, 11].

One response to this has been the Clinical Nurse Leader (CNL) education and practice initiative, which was introduced in the United States in 2003 by the American Association of Colleges of Nursing [12]. Since that time, numerous healthcare systems in the United States and abroad have implemented CNL roles and/or attempted to integrate CNL practice into their care delivery systems [13, 14]. While there have been studies demonstrating the effectiveness of the CNL role in improving patient quality and safety outcomes [15–17], other studies have shown that efforts to determine effectiveness have been hampered by ineffective implementation strategies and challenges to sustaining the role in practice [16, 18]. To help overcome these gaps in knowledge, a national level (United States) program of research focused on delineating a CNL care model was launched with the goal of producing a robust evidence base for CNL-integrated nursing care delivery [19, 20].

One outcome of this program of research has been an empirically validated explanatory model of CNL practice integration into frontline nursing microsystems [21]. The explanatory pathway for CNL practice integration incorporates multiple components including organizational readiness and CNL structuring elements that are theorized to influence CNL care model implementation, practice, and outcomes ([22, 23]; and next section for details). While case study data support the relationships proposed in the explanatory pathway [20, 24], to date only two studies have provided quantified representations of the relative importance of specified model components for successful CNL practice integration [25, 26].

Therefore, the purpose of this study was to identify and compare patterns of the operationalization of CNL care model domains in healthcare systems across the United States with measures of CNL implementation success. The specific aim of this study was to test the predictive nature of relationships between patterns of the operationalization of the CNL care model and implementation success. The overarching goal is to gain a deeper understanding of CNL practice implementation at the point of care and to better inform efforts to implement CNL practice using well-conceived and evidence-supported strategies that are empirically associated with higher levels of success.

## 2. A Description of the CNL Care Model

In the hospital setting, what many people think of when they think of a RN is a “staff nurse.” This is the RN who is assigned to a patient and is responsible for their care during their “shift,” which is typically either 8 or 12 h. This is the standard “nursing care delivery model” in hospitals, is called “primary care nursing,” and is operationalized as nurse-to-patient ratios based on patient acuity, i.e., how sick the patient is [27, 28]. This “RN staffing” model emphasizes the count of RNs that are required for a specific number of patients on a hospital unit per shift. For hospitals using 12 h shifts, RNs typically work three shifts per week. Hence, in this model, a patient is cared for by many staff RNs during the course of their hospitalization.

The CNL care model is a different way of organizing nursing care. Instead of focusing on RN ratios, the CNL care model uses CNLs at the unit level to lead the organization of patient care, leveraging RN and other clinicians' particular competencies and strengths, with the goal of providing consistently safe and high-quality care to patients [29]. Needleman [30] in a recent article has written about approaches to nurse staffing that move beyond the nurse-level staffing to consider “unit-shift level” staffing, which allows for flexibility in RN staffing and accounts for variability in nurse experience in any RN staffed shift. He cites the CNL model as one approach among others that serve as alternative approaches to safety-focused staff nursing models of care.

A CNL is an RN who receives a master's degree in an accredited CNL nursing program, allowing them to sit for and pass the national CNL certification exam, administered by the Commission for Nurse Certification. CNLs, in addition to retaining their RN competencies, graduate with three overarching “expert-level” competencies: clinical leadership, clinical outcome management, and care environment management [29]. Certification must be renewed every 5 years to continue to use the CNL designation.

In the CNL care model, staff RNs continue to work a certain number of 8–12 h shifts a week and focus on care for individual patients. CNLs are also structured at the clinical care unit level and typically there are 1-2 CNLs functioning within each unit. They are not responsible for administrative duties. While they can and do care for individual patients, their main responsibilities involve continual assessments of clinical unit functions to identify opportunities for clinical structures, clinical throughput, and clinical outcome improvement. They use their masters-level CNL competencies in clinical system leadership, clinical pathway/outcome management, and care environment/process management to (a) build relations with all clinicians entering the unit to care for patients, (b) facilitate effective communication and teamwork across clinicians focused on patient care, and (c) provide ongoing support to staff RNs so they can perform to their full scope of practice.

**Figure 1:**
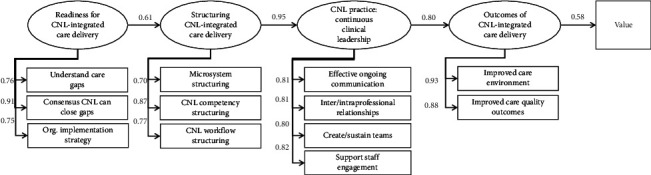
The validated CNL care model.

The validated CNL care model conceptualizes the model's structures and processes, incorporating 13 components organized into 5 conceptual domains of the care model: “Readiness for CNL integrated care delivery”; “Structuring CNL integrated care delivery”; “CNL Practice: Continuous Clinical Leadership”; “Outcomes of CNL integrated care delivery”; and “Value.” Previous studies using structural equation modeling empirically confirmed the directionality and significance of all hypothesized model pathways that establish the dynamics of action explaining how the CNL care model produces quality and safety outcomes [21]. The model was then operationalized into a 69-item CNL care model survey instrument and psychometrically validated using confirmatory factor analysis to affirm items express latent concepts of the CNL care model [20, 21]. See Figure 1 for the validated model.

## 3. Methods

### 3.1. Research Design

This study assessed the implementation of CNL care models across the Unites States in real-world situations and not as a manualized scientific experiment. The study used a cross-sectional research design to electronically administer the CNL care model survey instrument to a purposive sample of clinicians and administrators directly involved in their healthcare setting's CNL initiative.

### 3.2. Setting

The study setting involved a purposive set of clinical units in hospitals that have adopted the CNL care model. The study settings were purposefully selected by the AHRQ affiliate practice-based research network called the Clinical Nurse Leader Research Collaborative [19] for representative geographic region and setting ownership status diversity. The study only included hospitals that had redesigned their unit-level RN staffing model to integrate CNLs and excluded hospitals that had only integrated CNLs into the minority of their care delivery units or hired certified CNLs to work in traditional nursing roles (such as manager or educator). Health system champions have confirmed that, based on their own internal data audits, there were both relatively high performing (i.e., positive outcomes documented) and low performing (inconsistency or lack of positive outcomes) units across all 5 health systems, providing a spectrum of data needed for implementation and practice pattern analysis.

### 3.3. Sample

The exact population of clinicians and administrators (e.g., staff RNs, charge nurses, physicians, pharmacists, ancillary staff, managers, and executive administrators) directly involved in their healthcare setting's CNL initiative is unknown. All participants were recruited through network sampling [31]. Network sampling involves obtaining information from a specific community or group tied by a common relationship; in this study, the commonality was involvement with the CNL initiative. We knew the CNLs and study champions from the beginning so other members of the network were identified through these initial contacts. Other members of the network were identified through these initial network members. This network sampling method is considered a reasonable substitute for probabilistic sampling when the target population size is unknown. For complete details on sample recruitment, please see [32].

### 3.4. Measures

Data were collected using the psychometrically validated CNL care model survey instrument [20, 33] that includes subscales measuring the presence of the five domains of the CNL Practice model. Each domain is comprised of one to four components. Each component is measured with items (survey questions) numbering from 4 to 11 items per component (for details, see [33]) with a total of 69 items. Each item is rated by respondents as to the extent that item was present in their clinical setting using a slider bar from 0 (not present) to 100 (fully present). In addition to the items representing model domains and components, one survey item asks respondents to rate how successful the CNL initiative was in their clinical setting on a scale of 0–100, again using a slider bar. For complete details on survey development, see [21, 34].

### 3.5. Data Analysis

The survey data are complex, with three nested hierarchical levels, making it difficult to model using traditional frequentist approaches [35]. A Bayesian multilevel zero-one-inflated beta (ZOIB) regression model was therefore used to predict item level ratings by success score [36]. Item scores are bounded by a minimum (0) and maximum value (100), and hence treating them as proportions is more suitable than assuming they come from a normal distribution. Item and success scores were converted to proportions by dividing by their maximum value (100). ZOIB regression models can model a proportion on the [0, 1] interval, inclusive of the endpoints, by assuming the proportion comes from a mixture of distributions. A beta distribution is assumed over the (0, 1) interval, exclusive of the endpoints, while separate Bernoulli processes use two additional parameters to model the probability that a zero or a one occurs, and out of a zero or one, the probability that a one occurs. This mixture of distributions was especially important since many maximum and minimum ratings were expected in this setting.

To account for the fact that items are nested within components which are in turn nested within Domains (a domain/component/item hierarchical structure), models included varying intercepts and varying slopes for the effect of success score. Additionally, varying intercepts were included for each survey participant. Normal (0, 2.5) priors were used for the effect of success score on rating as well as for the standard deviations of all model parameters (intercept and slopes). A Student *t* (3, 0, 2.5), Beta (1, 1), and Gamma (0.01, 0.01) were utilized for the intercept, zero and one inflation parameters, and precision parameter (inverse of the variance), respectfully. For the correlation between the intercepts and slopes, a Lewandowski–Kurowicka–Joe (1) prior was used. The model was run in R Version 4.1.1 using the brms package [37] for Bayesian modeling and ggplot2 [38] for plotting.

### 3.6. Ethical Approval

Exempt human subjects' protection approval was obtained for this study through appropriate university and health organization Institutional Review Boards.

## 4. Results

### 4.1. Settings and Sample

See Table 1 for setting characteristics. Settings comprised individual units of hospitals within health systems, all of which were not-for-profit, with varying regional and national status.

See Table 2 for sample characteristics. Network email recruitment resulted in 1265 participants initially responding to the survey with 1223 (97%) providing success and rating scores. The sample included certified CNLs (11%) working in not-for-profit practice settings (100%). The majority of survey participants identified with a clinical practice role (87%), while 10% identified with an administrative or management role. The sample consisted of expert nurses with 16% having between 10 and 20 years' experience and 17% having over 20 years' experience as an RN.

### 4.2. ZOIB Regression Model

The model was run with 4 chains, each with 1000 burn-in iterations and 1000 posterior samples (for a total of 4000 posterior samples). An estimated 38.4% (38.1%–38.8%) of the ratings were either zero or one. Of the ratings that were either zero or one, 93.4% (93.1%–93.6%) were estimated to equal one, suggesting that ratings were skewed toward the upper bound of the maximal rating (i.e., a rating of 100). A total of 4127 (5%) of the 85,960 item responses that were missing across the 1223 participants was found to be missing at random and was simply accepted for complete case analysis.

### 4.3. Variability in Rating by Domain, Component, and Item

Table 3 provides estimates of the standard deviation and correlation of the intercept and success score at each level of the hierarchical structure, allowing for the identification of the hierarchy level that most discriminated between lower and higher success scores. Variability (standard deviation) in the baseline rating (rating at a success score of zero) was similar among domains, 0.30 (0.02–0.89), and among components within domains, 0.32 (0.17–0.57), as indicated by the standard deviation of intercepts for domain and components within domains (see Table 3). Variability in baseline rating at the item level (i.e., items within components) was lower, 0.24 (0.20–0.30), indicating that items within components were more homogenous in terms of rating than were components within domains or among domains. The variability of CNL care model element rating on implementation success scoring was greatest at the domain level, 0.55 (0.17–1.44), compared to either the component level, 0.28 (0.12–0.56), or the item level, 0.31 (0.25–0.39) (see Table 3). This indicates greater heterogeneity among domains for differentiation of rating by success score, compared to either the component level or item level. However, the strongest negative correlation, −0.93 (−1.00 to −0.67), was between the varying intercepts and varying slopes at the component level, suggesting that higher baseline (larger intercepts) ratings (where success score is zero) are correlated with lower values of the slope (i.e., less change in rating over the spectrum of success scores), and lower baseline (smaller intercepts) ratings are correlated with higher values of the slope (i.e., more change in rating over the spectrum of success scores). The marginal (average effect pooled across all participants and Domains/components/items) effect of a single unit increase in success score was a 2.79 (2.10–3.37) unit increase in the item-level rating on the logit scale (see Table 3).

**Figure 2:**
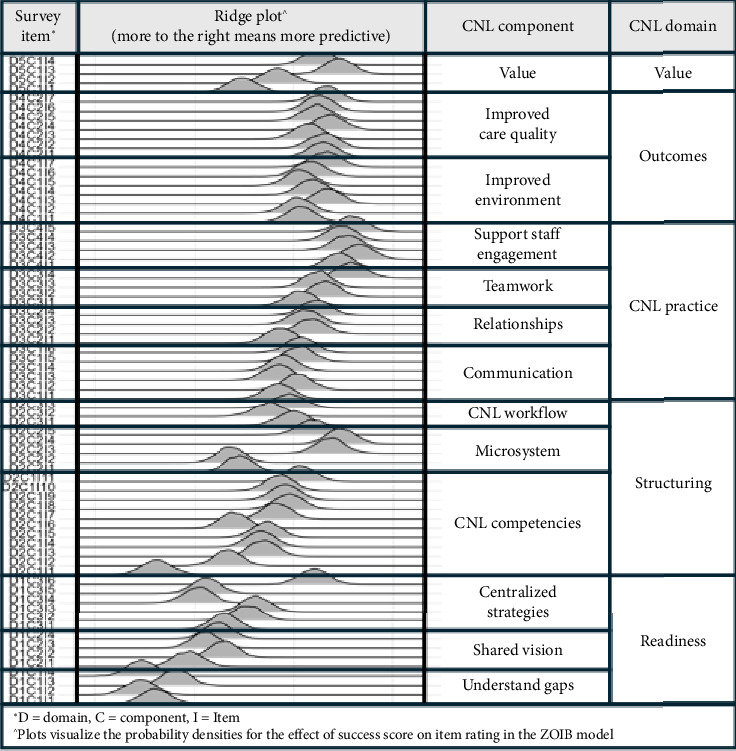
Probability distributions for the effect of success score and Clinical Nurse Leader (CNL) survey item rating, organized by CNL Domain and Component.

The posterior probability distributions for the effect of success score on rating for each model item (i.e., the posterior distributions for the varying slope for item) on the logit scale are presented in Figure 2. This figure includes ridge plots, which maps relative predictive strength from lower (toward the left) to higher (toward the right) effect size. The ridge plot allows the visual comparison of the probability densities for the parameter estimate for the effect of success score on item rating in the ZOIB model. The vertical axis provides the label for each item in terms of the nested structure of the model, for example D1C1I1 indicates Domain 1 Component 1 Item 1. The larger the value on the horizontal axis, the greater the change in item rating for every single unit increase in success score. In other words, larger values for the effect of success score indicate items that better discriminate between higher and lower success scorers.

### 4.4. Patterns of Prediction of Implementation Success

Overall, the ridge plot from this data analysis shows that most of the items in each domain have relatively similar effect sizes except for some exceptions in the Structuring and Readiness Domains and the Value Domain in terms of item ridge plot showing much lower or higher effect sizes within the item domain. Visually, most of the items indicating the higher effect score are in the CNL Practice and Outcomes domains of the CNL Practice Model.

Beyond the overall picture, the first specific visual pattern of note is the placement of the ridges corresponding to the items D3C4I1-5 and D3C3I4 farther to the right than the remainder of the ridges in the plot (see Figure 2). These items are in the CNL Practice domain, specifically in the Support Staff Engagement and Teamwork components. Placement of these items farthest to the right indicates that these items have relatively stronger predictive relationship with respect to implementation success than any other elements in the CNL practice model. The five items in the Support Staff Engagement component are “CNL provides ongoing support for staff to lead their own practice,” “CNL is a consistently present role model for all staff working at the point of care,” “CNL is a responsive/available resource to staff based on their needs at the moment,” “CNL helps staff identify and create solutions for patient care needs,” and “CNL empowers staff nurses to perform to their full scope of practice.”

A second pattern of note is the placement of two ridges (D2C2I3 and D2C2I4) in the Microsystem Structuring component of the Structuring domain farther to the right than any of the other ridges in the plot, with the exception of the practice items discussed in the previous paragraph. These two items with greater relative predictive strength are “CNL is consistently present at the point of care” and “CNL practice is aligned with frontline clinical care delivery needs.” Item D2C2I5 within this component, which is “CNL practice is aligned with executive quality and safety priorities,” also exhibits relatively higher predictive strength.

A third pattern is the appearance of several ridges corresponding to items in the domains of Readiness and Value farther to the right of the plot. First, the ridge corresponding to item D1C3I6 is farther to the right than any other ridges in the Readiness domain, and more in line with the predictive strength evidenced by the items in the CNL Practice and Outcomes domains. This single Readiness item is “There is a shared vision for CNL practice at the executive, department, and point of care level.” Secondly, the items D5C1I3 and D5C1I4 demonstrate relatively greater predictive strength than about half of the other items. These two items are “CNL practice is valued by point of care staff” and “CNL practice is valued by point of care multiprofessional clinicians.”

The final visual pattern of interest is the consistent placement of the majority of the items comprising the Outcomes domain on the ridge plot. This pattern indicates the importance of the overall relationship between outcome achievement and successful implementation, but also illustrates that all of the outcomes specified in the CNL Practice Model are roughly of equal importance as indicators of CNL practice integration. The elements corresponding to CNL Outcomes are listed in Table 4 and will be discussed in relation to CNL care model implementation in more detail in the following section.

## 5. Discussion

The Bayesian multilevel modeling approach was employed with the survey data to gain a better understanding of how the implementation of a well-specified, but complex, nursing care delivery model directly influences successful adoption into practice. The design and approach of this inquiry do not assume an idealized conceptualization that standardized implementation happens unproblematically as a matter of course, nor that implementation happens homogenously in diverse systems and clinical units. The importance of an adequate implementation-before-effectiveness trajectory has been demonstrated in previous studies [13, 20, 25] and a CNL evidence review [16], showing that the ways in which the CNL care model is implemented significantly influences whether or not it will achieve expected quality and safety outcomes.

A recently published paper using data from a 2015 survey of a Unites States national-level sample of certified CNLs [26] also employed a Bayesian multilevel modeling approach to analyze real-world data pertaining to CNL practice adoption. The 2015 dataset comprised a nation-wide sample of certified CNLs and clinicians/administrators self-identifying as involved in the implementation of a CNL initiative. More than 80% of this sample identified as a certified CNL [26] but only 67% reported practicing in a formal CNL role. Furthermore, only 38% of all the total respondents were reporting on an “established” CNL care model, while the majority of respondents (76%) were reporting on the developmental stages of implementation in their clinical setting.

This is in contrast to the current study, which involved participants of health settings that had actively established the CNL care model across their hospital units and were in the stage of formative and summative evaluations of the model (see Table 2). The context and conditions for the previous study were at a time in the history of the CNL initiative when individuals and systems were attempting to adopt CNL practice without a validated roadmap for guidance. The yardstick for success in the earlier time period was largely focused on getting CNL roles designated and/or established somewhere in the organization compared to the objective of the healthcare systems in the current study to adopt and evaluate a CNL integrated model as the frontline model of care delivery [39]. Findings of the current study, and consideration of findings from the analysis of the previous 2015 dataset, provide analytic specificity about which operationalization components most influence implementation success and enhanced understanding of the interdependency of context and pathway to success, which is discussed in the following sections.

### 5.1. Organizational Readiness

Organizational Readiness (Domain 1 of the model) is conceptualized through three components: understanding care delivery gaps, consensus that the CNL care model can close gaps, and an organization-level implementation plan is developed and followed. Across these components, a single item in the organization-level implementation strategy component stood out as most predictive of implementation success. The item (D1C3I6 in Figure 2) is “there is a shared vision for CNL practice at the executive, department, and point of care level.” It seems reasonable to suggest that without a shared vision for the CNL role at the level of frontline practice and meso-to-macro level administrative leadership, implementation will not succeed.

People working across these different levels might have very different ideas about what a change to a CNL care model might achieve. A chief nursing officer may feel the model can impact regulatory outcomes and nursing sensitive indicators and want to structure CNL role accountabilities with that in mind. A department or service line manager may have very specific clinical indicators that need addressing and want to structure CNL role accountabilities with those in mind. Staff nurses, who know the patients and care barriers best, have their own ideas about how these gaps can be addressed through care assistance and process streamlining. These are all very different visions of CNL role accountabilities, and if they cannot come together, as previous CNL case studies have shown, these conflicting ideas can stop a CNL initiative in its tracks (see, for example, [24]).

It is interesting to note that in the previous Bayesian study [26], which used the same survey and Bayesian analytic procedures but in a different sample, the items in the organization-level strategy of the Readiness domain exhibited the most predictive capacity. At that stage of the CNL initiative, with success largely represented as getting a CNL role formally established, it emerged as a critical consideration of readiness as an antecedent to merely establishing a CNL position. In this context, the shared vision for CNL practice was representative of a necessary step for obtaining adequate buy in across key stakeholders. The similarity in predictive capacity across the two datasets of the items “having a shared vision,” “a global strategic plan to reorganize care delivery to include CNL practice,” and “an appropriate change management strategy” implicate these critical components of readiness in creating optimal antecedents to successful implementation.

The lower predictive strength of the other two components (Understanding care delivery gaps and Consensus that CNL model can close gaps) in the Readiness domain in the current study, which emerged as relatively more predictive in the 2015 sample [26], seems to indicate that those components alone would not provide predictive threshold without operationalization of the items specified in an organization-level implementation strategy. Differences in the relative predictive strength associated with Readiness in this study's sample as compared to the most predictive strength in the 2015 sample may also reflect the importance of contextual variables like the evolution of knowledge over time about CNL practice and the timing of survey response with respect to the lifecycle of the CNL initiative about which the respondents were replying to survey items. In the current study, the respondents in healthcare systems that had implemented CNL integrated care delivery may not have been as aware of the foundational work done to get the initiative to where it was at the time of the survey (63% participants reported their responses were from after the model was implemented, i.e., established).

### 5.2. Structuring

CNL Structuring (Domain 2 of the model) is conceptualized in the CNL Practice model through three components: CNL-level structuring, microsystem-level structuring, and workflow-level structuring. The items in these components specify that: CNLs must be competent in their master's level knowledge, skills, and abilities to be able to perform adequately; the microsystem level nursing staff model is adequately reorganized to integrate CNLs; and CNLs are accountable for their daily workflow involving clinical leadership, clinical outcome management, and care environment management. In the current study, although most of the Structuring items were less predictive than the items specifying the domains of CNL Practice, Outcomes, and Value, there were two Structuring items that were more predictive of implementation success than all but four of the items in the CNL Practice domain. The two structuring items are D2C2I3 “the CNL is consistently present at the point of care (i.e., where the patients are)” and D2C2I4 “CNL practice is aligned with frontline clinical care delivery needs.”

Interestingly, the pattern of relative predictive strength of the items in the CNL microsystem structuring component in the 2015 sample is very different. The five Items in this component in the earlier study were more equally predictive of success and were among the items most predictive across the entire model. The lesser relative predictive strength of items “nurse staffing model is reorganized to integrate CNL practice” and “CNL practice involves minimal administrative management duties” in the current study may again reflect the timing of the survey in the lifecycle of the initiative. Care model reorganization and designation of CNL duties, while important to success, may be less visible to survey respondents in the current study in the context of everyday practice that has integrated CNL practice, shifting importance to the consistent presence and alignment of practice with frontline needs and quality/safety priorities. This finding, and the influence of context on implementation pathways, is reinforced by earlier anecdotal reports of barriers to CNL role implementation that included CNLs being “pulled from” the point of care or “pulled into” administrative duties [25].

### 5.3. CNL Practice

CNL practice is conceptualized in the CNL care model as a downstream manifestation of sufficient attention to readiness and adequate/appropriate structuring. The nature of CNL practice is conceptualized via the four components of communication, relationships, teamwork, and supporting staff engagement. In the current dataset, scores of the items most predictive of implementation success overall were items of the CNL Practice domain component “Supporting Staff Engagement.” The items include “the CNL provides ongoing support for staff to lead their own practice,” “the CNL is a consistently present role model for all staff working at the point of care,” “the CNL is a responsive/available resource to staff based on their needs at the moment,” “the CNL helps staff identify and create solutions for patient care needs,” and “the CNL empowers staff nurses to perform to their full scope of practice.”

What this suggests is that CNL practice with respect to frontline staff within the frontline context of practice is a key marker of implementation success. A pattern of success emerges from the data suggesting that successful implementation of the CNL care model is grounded in a shared vision for CNL practice that is consistently present at the point of care, aligned with both frontline needs and quality/safety priorities, and focused on supporting frontline staff in their practice. The items specified in the model for this component of practice delineate provision of active and continual clinical assistance to staff nurses on a daily basis that goes beyond simply assuming portions of workload or tasks. Items inclusive of supporting staff to lead their own practice and empowering staff to perform to their full scope are representations of leadership. Helping staff to identify and create solutions for patient care needs manifests from an ongoing engagement through which the CNL becomes aware of care barriers prompted by staff RNs or other clinicians, and even barriers that may be invisible to staff RNs and clinicians who are not present on the unit as consistently as the CNLs. The CNLs work with staff RNs and others who are involved with a particular care process to improve the process holistically, i.e., considering downstream and upstream perspectives as well as the unit-level perspective. Through CNL practice, guided by CNL accountabilities (clinical leadership, clinical outcome management, and care environment management), frontline staff knowledge is strategically leveraged to make a difference at the frontlines themselves for the benefit of clinicians and patients.

The importance of this component (Supporting staff engagement) of CNL practice in the success of implementing the CNL care model was not evident in the 2015 sample. In general, the items representing CNL practice in the 2015 sample had less predictive power than the items in the Readiness and Structuring domains. The aspect of CNL practice that was most predictive in the 2015 sample was teams and teamwork, which involves bringing people together to collaboratively improve care processes. This difference between the current study sample and the 2015 sample most likely reflects the earlier “project-focused” orientation that certified CNLs initially adopted to help create the business case for creating CNL roles as compared to the integration of CNL practice into frontline care delivery that manifests in the current study. This finding of the predictive strength of CNL practice integrated into the frontline care model attests to the initial vision of the CNL role as a member of the microsystem care team [12].

### 5.4. Outcomes

As described previously and visualized in the ridge plot (Figure 2), the predictive strength on implementation success of the items across the two components of the Outcomes domain was consistently and relatively high. Again, it is important to revisit how outcomes are specified in the model items as representations of conditions for professional practice and optimization of quality and safety outcomes, rather than numeric values of identified performance or quality measures (see Table 4). The inference is that the CNL care model, successfully implemented, creates conditions for improved care environments and improved patient care quality. While evidence from previous studies specifically analyzing care quality metrics demonstrated improvements in outcomes such as patient satisfaction and reduced variability in nursing sensitive quality and safety outcomes [17, 19], those measures are, to some extent, downstream indications of the influence of the CNL care model on care coordination, reduction of gaps in care, prevention of errors reaching the patient, and staff spending more time with patients.

The findings suggest that the CNL care model is considered successful when it is implemented in a way that the frontline clinical staff perceive concrete improvements in their care environment. Items corresponding to improved care environments include “there are effective communication processes across professions,” “staff feel like they own their practice,” “multiprofessional clinicians regularly collaborate to plan patient care,” “physicians are more satisfied with the care their patients receive,” and “multiprofessional clinicians regularly work together to solve clinical problems.” Equally important measures of outcomes specified in the improved care environment component of the Outcomes domain would recognize and connect effective communication, ownership of practice, multiprofessional collaboration, multiprofessional problem solving, and improved dynamics of clinical interactions with improved quality outcomes.

The Outcome domain in the 2015 sample was similarly consistent across items but did not emerge as a domain with relatively higher predictive strength in terms of implementation success. While it was a bit unnerving at that time to think that Outcomes might not be important to successful implementation, especially in a climate characterized by demands for tangible reductions in cost and improvements in performance measures for the CNL business case, it becomes apparent in retrospect that the outcomes specified in the model were largely not the types of outcomes that were used to make the business case argument for CNLs when it was first being adopted [40]. Then, CNLs and nursing leaders were largely relying on performance indicator improvements and cost reduction calculations from individual CNL led improvement projects to achieve the success yardstick of getting a CNL role formally designated in the organization [41].

### 5.5. Value

As already noted, the outcomes attributable to successful CNL care model implementation are related to the influence of adequately structured CNL practice on care coordination, reduction of gaps in care, prevention of errors reaching the patient, and staff spending more time with patients. In the current analysis, two items in the Value domain exhibited predictive strength equivalent to the predictive strengths of items in the Outcome and CNL Practice domains. The item “CNL practice is valued by point of care staff” was closely followed by “CNL practice is valued by point of care multiprofessional clinicians.” This finding reinforces the inference that successful implementation of the CNL care model manifests as CNL practice located within, aligned with, and focused on the frontlines of care delivery with strategic intent to create conditions for professional practice and optimized quality/safety outcomes. It is these practices and outcomes that staff and multiprofessional clinicians value, and which are predictive of successful implementation, in terms of the model is functioning as it was conceived.

Importantly, this conception of the CNL care model needs to be shared with executive leaders as well as frontline clinicians. The model will be valued to the extent that it delivers (in terms of structures, practices, and outcomes) on the vision developed in the Readiness stage of implementation. This is reflected in the other items in the Value domain: “CNL practice is valued by executive leaders” and “CNL practice is valued by point of care/department managers.” Although slightly less predictive of success, these items represent two significant barriers that have already been cited in reports of unsuccessful CNL implementation. What was found was that executive and management stakeholder commitment to the model needed to be sustained past initial phases, in terms of responding to challenges and barriers revealed in “pilot” rollouts, in order for implementation to eventually become successful [25].

It is the leaders and managers that control the resources and supports allotted to the model's implementation. Based on the findings of the current study, it can be hypothesized that the extent to which a shared vision was developed by both administrative leaders and frontline clinicians is a driver of the willingness to redirect resources and adapt implementation strategies to contextual realities. This shows up “downstream” as value placed on the model as a whole. The temporal relationship of model domains suggests that readiness carries through to structure and practice implementation, which supports expected outcomes. This confirms the initial shared vision and results in positive valuation of the model by both leaders and clinicians.

## 6. Implications

Study findings align with growing evidence in the implementation science field that contextual elements of the implementation of any efficacious intervention directly influence its adoption into real-world healthcare settings, or as Damschroder and colleagues state in their article on the updated Consolidated Framework for Implementation Research (CFIR), “implementation science embraces the reality that contextual factors are active and dynamic forces working for and against implementation efforts in the real world” [42]. The updated CFIR acknowledges that elements such as local attitudes and conditions, leadership engagement, available resources, and implementation teams are constructs that directly influence the adoption of evidence into practice. Thus, it is noteworthy that the CNL care model not only incorporates these elements into the model, but that this study and others have found that the degree to which these constructs have been operationalized has a direct influence on implementation success and the accrual of expected outcomes.

Furthermore, it is important to note that one of the outcomes expected with successful CNL care model implementation is improved care environment. We have discussed how each domain of the model had as its most predictable marker of implementation success the idea of a focus on the frontline care setting, from executive leaders needing to be aligned with staff RNs about the importance of focusing on the clinical unit, to ensuring CNLs are positioned at the clinical unit, to the need for CNLs to focus their workflows and practices on emerging unit-level issues with care trajectories. It is this central focus, that practices are oriented to identifying and solving problems that frontline clinicians themselves articulate, that seems to be the common element across each domain driving the outcomes of improved care environments and care quality.

This outcome and the implementation patterns we have addressed that are predictive of implementation success and outcome achievement stand as an important finding in the current healthcare context. The National Academy of Sciences, Engineering, and Medicine (NASEM), in its 2024 publication “National plan for health workforce well-being,” specifies the creation of positive work environments as the first goal in achieving workforce well-being. Positive work environments are posited as the solution to the current crisis in hospital contexts related to clinician burnout. While burnout manifests in individuals, its root causes lie within the work environments clinicians work in.

This is why NASEM prioritizes a systems-level approach to the problem and calls for the “redesign [of] how health is delivered” ([43], p. 5). While they do not provide any blueprints for achieving this redesign, they describe critical elements, including “improve health worker and learner well-being in strategic plans … establish mentorship programs to help all health workers … provide mechanisms and systems to allow health workers to operate as teams … learn about health worker experiences directly by asking them … allocate the resources necessary to implement strategies that will improve health worker well-being … [and] evaluate demonstration programs and grants in the workplace” ([43], Goal 1, actions 1.1.A, 1.2.D, 1.3.A, 1.3.H, 1.4.D, and 1.5.B).

The CNL practice components of facilitating communication, strengthening interprofessional relationships, building teams and effective teamwork, and supporting staff engagement are core practices that align with and address critical elements of the NASEM framework. Furthermore, the CNL care model includes seven items expressing improved care environments resulting from CNL practice implementation. Importantly, these seven items align robustly with NASEM stated characteristics of positive work environments. For example, The NASEM work environment element “facilitates participatory decision making” can be directly correlated to the CNL care environment factors “multiprofessional clinicians regularly collaborate to plan patient care” and “multiprofessional clinicians regularly collaborate to solve clinical problems.” This means the CNL care model comprises an evidence-informed and system-level approach to improving positive work environments by bringing about the characteristics and climate described by NASEM. The CNL care model can address the healthcare workforce crisis by directly addressing each of the NASEM action items listed.

## 7. Recommendations for Practice

Important considerations emerge from this program of research examining relationships between implementation patterns of CNL practice in a hospital context and implementation success that can serve as guidelines for systems endeavoring to integrate, sustain, and/or evolve a CNL care model. Importantly, much practice-based evidence has already been disseminated by health system “pioneers” who have written of their implementation experiences, including strategies used to gain readiness for and implement the CNL care model into their clinical microsystems. What the current study does is inform these specific strategies with evidence rationalizing their targeted implementation domains. These targeted domains include (1) creating a shared vision reflective of context, (2) following a strategic approach with attention to effective change management, and (3) effectively operationalizing CNL competencies into CNL practice responsive to point-of-care context in real time.

In terms of creating a shared vision and following a strategic approach, Williams et al. [41] describe the Veterans Administration's (VA) “Clinical Nurse Leader Implementation and Evaluation Service,” which worked across the VA system to help hospital nurse leaders strategically approach CNL practice integration, including creating a shared vision, identifying necessary resources and management tools, and adapting the CNL care model based on continuous monitoring. In terms of effective change management, another regional health system that implemented the CNL care model (see [19] for details) described how the data they captured on their CNL implementation efforts were specifically used to improve their Readiness resources, which included a new CNL orientation structure and an orientation process to be used within leadership to clarify CNL practice with the goal of creating a shared understanding of the model and CNL role. Finally, another set of papers describe the importance of a commitment to CNL practice development as a mode for ensuring a strong “novice-to-expert” trajectory for certified CNLs hired into the role [18, 44, 45]. With the novelty of the CNL care model, the papers demonstrate the need for, and provide concrete strategies targeting, active support for CNLs as they situate themselves into clinical microsystems, gain the appropriate microsystem perspective, identify microsystem patterns needing improvement, and apply their communication and teamwork competencies to effect change in a collaborative manner.

The findings from this study corroborate these strategies as focused on predictable implementation “roadblocks” at the Readiness, Structuring, and CNL Practice stages of the CNL model adoption. While these strategies were not rigorously tested using appropriate research designs and methods, we feel this is not a barrier to their use or application, since each was developed specifically based on their settings' given realities, which are described in great detail in the papers, and which health systems can compare and contrast with their own given realities and adapt where necessary.

## 8. Limitations and Conclusions

The complexity of care delivery and the cost associated with changing care models for research purposes mean an experimental design was not possible for this program of research. While we have overcome this issue by taking advantage of health systems that have implemented the CNL care model, it is noted that the current study is observational in nature. The results however align with other quasi-experimental CNL studies (e.g. [15]) and help to explain those study's findings in terms of how CNL implementation and practice can improve care quality outcomes, although it is acknowledged that more research is needed to strengthen overall causal claims. This includes future (planned) research that provides specific analyses of the relations between implementation patterns and specific unit characteristics (e.g., unit patient population), which can help determine which contexts benefit most from CNL implementation and contextual characteristics that facilitate or impede CNL implementation success. This also includes future research to determine whether the CNL care model, designed as an advanced generalist nurse embedded into a clinical team functioning at the frontlines of care in the hospital setting, can be adapted to function outside the hospital setting, such as an ambulatory clinical or public health setting. Previous research has identified that CNLs are present in ambulatory settings [13], but not much is known about their functioning within ambulatory care teams.

Despite (or perhaps because of) these limitations, this study (and program of research) is innovative in how it approaches nursing care delivery and its measurement. The evidence is clear that RNs influence patient quality and safety outcomes. *What remains unclear is how to organize and implement nursing knowledge and practice into care delivery models that consistently achieve quality and safety mandates*. Nursing care delivery models are complex, dynamic, and inherently context sensitive. There is growing consensus in the implementation and health services research fields that inquiry into, and evaluation of, complex healthcare delivery systems must move past traditional binary questions of efficacy and toward a more sophisticated exploration of “generalizable determinants of beneficial outcomes,” which include implementation strategies that facilitate adoption and success [46–48].

This study began with a conceptually clear understanding of the CNL care model that delineates a complex causal pathway toward effectiveness. In terms of methods, a focus on implementation was critical because it provides necessary information about what ways and to what extent the model has been adopted in diverse clinical settings, as well as determining the model's overall effectiveness. To our knowledge, this program of research involves the first large-scale implementation-effectiveness examination of any nursing care delivery model, and through meticulous preliminary research, has benefited from tools with which to delineate how CNLs have been implemented into redesigned care delivery systems and to determine how this implementation influences CNL practice and health outcomes.

Based on this study and other comparable research, CNL readiness and structures can now be quantitatively linked to measures of CNL implementation success in a way that can identify and account for variability in CNL implementation and its influence on quality and safety outcomes. These relationships provide detailed and actionable evidence about what works, how, and why it works in terms of nursing practices and their contexts, and toward what consistent measurable outcomes. Findings provide critical evidence for nursing-led changes to clinical health systems in ways that produce positive work environments as well as improved care quality, thus offering nursing-led solutions for today's urgent crisis in health workforce well-being.

## Figures and Tables

**Table 1:** Study health system setting demographics.

Health system	# study hospitals in system	# study units across hospitals	Region
Community hospital within national not-for-profit health system	1	11	Midwest
Community hospitals within a state-wide non-for-profit health system	3	15	Southeast
Community hospitals within a large regional not-for-profit health system	3	21	Southwest
Community hospital within multiple-state not-for-profit health system	1	13	East
Community hospital partnered with a university medical center	1	6	Midwest

**Table 2:** Study sample demographics.

Characteristics of sample	Count (*n* = 1223)	Percent
Education level (more than one answer possible)		
Associate	186	15
Bachelor	711	58
Masters	344	28
Doctorate	105	9
Years as registered nurse (RN)		
Less than 5 years	209	17
5–10 years	235	19
11–20 years	199	16
Over 20 years	212	17
CNL certified (yes)	130	11
Current primary role		
Clinical practice	1058	87
Education	15	1
Administration/management	128	10
Other/unknown	22	2
Unit type		
Emergency department	71	6
Intensive care unit	68	6
Labor and delivery/obstetrics	27	2
Medical surgical	433	35
Progressive care	57	5
Other	135	11
Phase of CNL initiative		
Preimplementation	104	9
Implementation	215	18
After implementation	770	63
Other/unknown	182	15

**Table 3:** Bayesian multilevel model effects.

Level effects	Estimate	95% confidence interval
*Group level effects*		
Domain (*n* = 5)		
sd (intercept)	0.30	(0.02, 0.89)
sd (success)	0.55	(0.17, 1.44)
cor (intercept, success)	−0.64	(−1.00, 0.66)
Component (*n* = 13)		
sd (intercept)	0.32	(0.17, 0.57)
sd (success)	0.28	(0.12, 0.56)
cor (intercept, success)	−0.93	(−1.00, −0.67)
Item (*n* = 70)		
sd (intercept)	0.24	(0.20, 0.30)
sd (success)	0.31	(0.25, 0.39)
cor (intercept, success)	−0.84	(−0.91, −0.74)

*Population level effects*		
Intercept	−1.17	(−1.57, −0.74)
Success	2.79	(2.10, 3.37)

*Note:* cor = correlation.

Abbreviation: sd = standard deviation.

**Table 4:** Outcomes specified by items in the outcomes domain of the CNL practice model.

CNP care model outcome domain components	Items expressing CNP care model outcome domain components
Improved care environments	There are effective communication processes across professions
	Staff feel like they own their practice
	Staff are more satisfied with the care environment
	Multiprofessional clinicians regularly collaborate to plan patient care
	Physicians are more satisfied with the care their patients receive
	Multiprofessional clinicians regularly work together to solve clinical problems
	CNL practice changes the dynamics of clinical interactions between multiprofessional clinicians or the better

Improved care quality	Patients and families experience improved care coordination
	There are improvements in point of care nursing sensitive quality indicators
	There is improvement in national quality benchmark outcomes
	There is improved care coordination
	There are less gaps or omissions in care
	Errors are prevented/caught before they reach the patient
	Staff spend more time with patients

## Data Availability

The data that support the findings of this study are available from the corresponding author upon reasonable request.
